# Clinical Guidance for Lipodystrophy Syndromes: From Diagnosis and Work-Up to Treatment

**DOI:** 10.1007/s11892-025-01603-4

**Published:** 2025-09-02

**Authors:** Donatella Gilio, Maria Foss-Freitas, Elif A. Oral

**Affiliations:** 1 Metabolism, Endocrinology and Diabetes (MEND) Division, Internal Medicine Department, University of Michigan, 2800 Plymouth Road Building 25, Room 3696, Ann Arbor, MI 48105 USA; 2 Department of Clinical and Translational Sciences, University of Pisa, Pisa, Italy

**Keywords:** Lipodystrophy, Adipose tissue, Metabolic abnormalities, Phenotype, Insulin resistance, Leptin

## Abstract

**Purpose of Review:**

The goal of this review is to address the challenges in diagnosing and managing lipodystrophy syndromes.

**Recent Findings:**

Clinical and metabolic assessments, along with genetic analyses, are essential for tailoring medical care and providing appropriate genetic counseling. Efforts are underway to develop more objective diagnostic tools using imaging techniques or novel biomarkers. Leptin therapy has been a significant breakthrough for generalized lipodystrophy treatment; however, more effective treatments are still needed for partial and acquired forms. While gene editing and transcript modification strategies are being explored for specific forms of lipodystrophy, reducing the burden on adipocytes by lowering caloric intake remains a fundamental approach across all forms of the condition. As supporting data emerge, agents that reduce caloric intake may become integral to treatment algorithms.

**Summary:**

This review offers practical guidance for clinicians managing patients with lipodystrophy, highlighting advances in diagnosis, treatment, and ongoing challenges in clinical care.

## Introduction

Lipodystrophy syndromes are a heterogeneous group of rare diseases characterized by varying degrees of fat loss. These disorders can be categorized into two etiological groups: hereditary and acquired forms. They are further distinguished by the pattern of adipose tissue loss, which can manifest as either a widespread absence of fat throughout the body (generalized lipodystrophy) or a depletion of adipose tissue in specific regions (partial lipodystrophy) [1]. The metabolic consequences of lipodystrophy can be severe, with insulin resistance being a hallmark feature, often leading to significant health complications. However, the clinical manifestations of lipodystrophy syndromes are remarkably diverse, presenting a considerable challenge for healthcare professionals in terms of accurate diagnosis and subtype classification [2–5]. Our goal in this review is provide practical guidance to clinicians who may encounter these conditions.

### Diagnosis (Fig. 1)

**Fig. 1:**
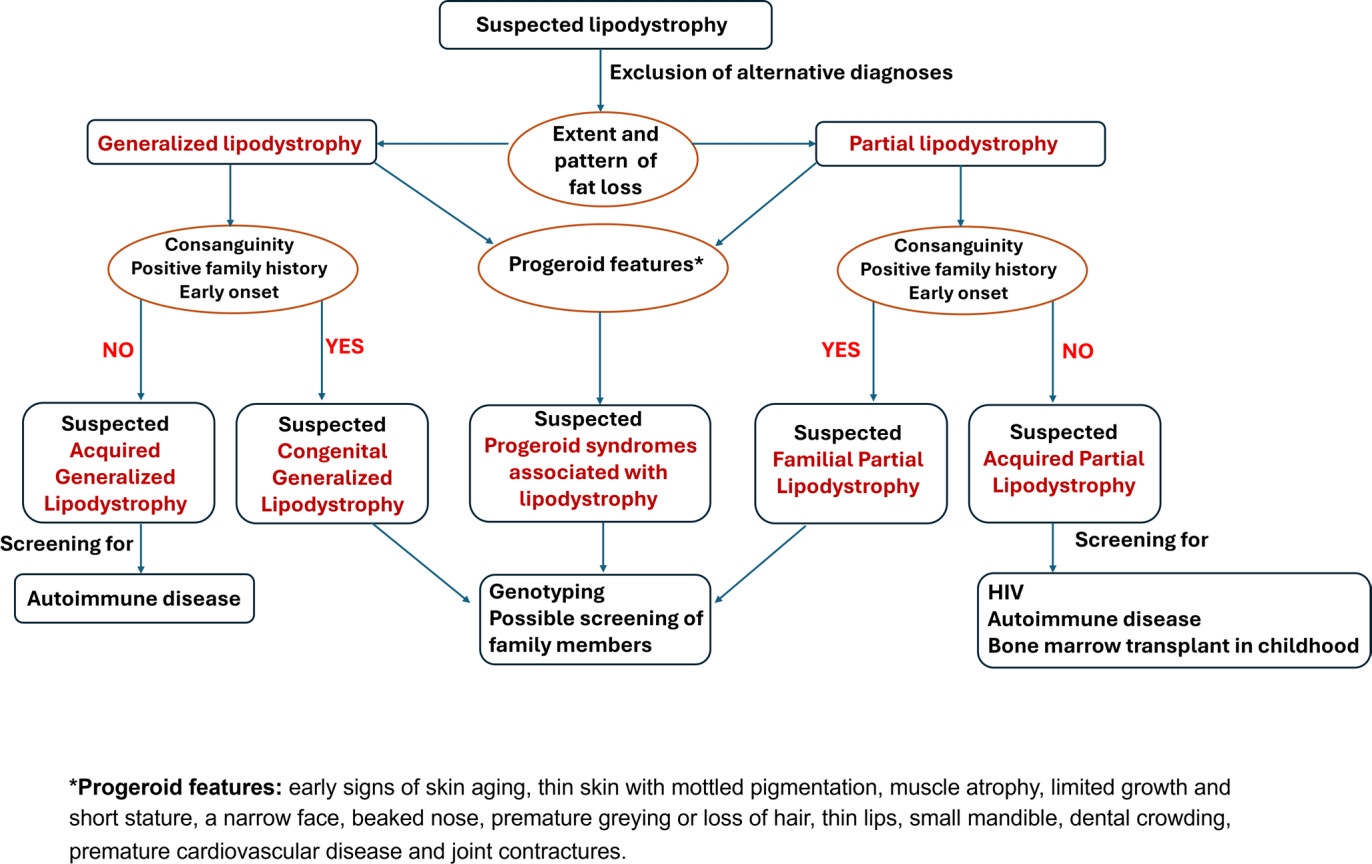
A clinical algorithm for approaching a patient presenting with suspected lipodystrophy

The diagnosis of lipodystrophy is challenging due to the lack of well-defined diagnostic criteria, making it dependent on a combination of factors, including the patient’s medical history, patterns of fat distribution, physical examination, presence of metabolic complications, imaging studies, and other clinical indicators unique to each subtype [1]. This approach highlights the importance of a comprehensive evaluation in the absence of standardized guidelines.

### Clinical Evaluation as the First Diagnostic Step

Any individual with partial or complete loss of subcutaneous fat should be evaluated for lipodystrophy. Comorbid conditions such as diabetes mellitus (DM), severe hypertriglyceridemia with or without acute pancreatitis, polycystic ovary syndrome (PCOS), or metabolic dysfunction-associated steatohepatitis (MASH) would raise clinical suspicion for lipodystrophy [1].

Generalized lipodystrophy (GL) typically presents with a more recognizable and pronounced phenotype, characterized by the complete absence of adipose tissue (AT) and a muscular appearance. In contrast, partial lipodystrophy (PL) often presents more subtly, with distinctive patterns of fat loss and accumulation [6–8]. When a newborn, infant, or young child displays a muscular appearance and a near-total, generalized lack of body fat without any signs of malnutrition or a catabolic state, lipodystrophy might appear to be an obvious diagnosis. However, the condition is often not recognized at birth, leading to delayed diagnosis due to its rarity and low awareness among healthcare providers.

When GL is diagnosed, the first step should be to gather more clinical data to differentiate between congenital and acquired forms. A detailed medical history is essential for determining the age at which fat loss began. In congenital generalized lipodystrophy (CGL), fat loss is typically noticeable shortly after birth or within the first few years of life. In contrast, acquired generalized lipodystrophy (AGL) usually manifests in late childhood or early adulthood, though rare cases presenting in the 6th and 7th decades of life have been observed in our clinical setting (authors’ unpublished data). Photos from early childhood can help distinguish between these two forms. CGL often involves increased appetite and accelerated growth during early childhood [9], whereas AGL is frequently associated with autoimmune disorders. Due to the pronounced loss of adipose tissue, patients with GL may be misdiagnosed with anorexia nervosa or malnutrition. Patients with CGL may exhibit acromegaloid features, but the absence of subcutaneous adipose tissue (SAT) differentiates it from acromegaly [10–12], and low to normal IGF-1 levels and glucose-responsive GH levels further support the diagnosis [13, 14].

Clinical diagnosis can be more challenging for PL. In women with familial partial lipodystrophy (FPLD), the diagnosis is often aided by the selective absence of fat, along with a high prevalence of hirsutism, menstrual irregularities, and metabolic abnormalities [15, 16]. Diagnosis can be more difficult in men due to their more subtle phenotypes, and a less aggressive metabolic profile compared to women [17].

The differential diagnosis for FPLD may include Cushing’s syndrome due to the presence of features such as dorsocervical fat pad and moon facies. However, patients with FPLD lack typical Cushing’s signs like muscle weakness and purple striae [2, 6]. Serum cortisol levels and the dexamethasone suppression test are also crucial for differential diagnosis.

Clinical features such as early signs of skin aging, thin skin with mottled pigmentation, muscle atrophy, limited growth and short stature, a narrow face, beaked nose, premature greying or loss of hair, thin lips, small mandible, dental crowding, premature cardiovascular disease and joint contractures should raise suspicion for progeroid syndromes [18].

Pedigree analysis may suggest genetic forms of lipodystrophy, and parental consanguinity should heighten suspicion for autosomal recessive disorders. Conversely, autoimmune conditions and the use of certain medications, such as HIV antiretrovirals, corticosteroids, checkpoint inhibitors and insulin, can suggest acquired forms of lipodystrophy [1].

Patients should be screened for clinical characteristics of lipodystrophy subtypes, metabolic complications and comorbidities. Skin features like acanthosis nigricans and hirsutism in women may indicate insulin resistance, while eruptive xanthomata and lipemia retinalis suggest severe hypertriglyceridemia. Ophthalmic exams can reveal complications, such as diabetic retinopathy, or subtype-specific signs like retinal drusen in patients with Barraquer-Simons syndrome.

### Anthropometry and Body Composition

A physical examination is crucial for providing evidence of decreased fat. In individuals without lipodystrophy, SAT is always palpable upon careful examination. While skinfold measurements can be useful, they are not standardized. Skinfold values below the 10th percentile increase suspicion but are not diagnostic. In our experience, mid-thigh skinfold cut-offs of 11 mm in adult men and 22 mm in adult women can support diagnosis in the European, Middle Eastern, North African and South American patients. However, similar cut-offs should be derived for Southeast Asian and Asian populations. The KöB index (subscapular/calf skinfold ratio, with a cut-off of 3.477) also helps distinguishing FPLD1 from androgenic obesity in women [19].

In patients with clinical suspicion of lipodystrophy, body composition analysis using dual-energy x-ray absorptiometry (DXA) and whole-body magnetic resonance imaging (MRI) can further support diagnosis [1, 20]. These techniques are increasingly available in clinical settings, although their interpretation in the context of lipodystrophy may still require specialist expertise. Our group developed a “fat shadow” technique using DXA images to visualize fat distribution, which showed 100% sensitivity and specificity for GL and 85% sensitivity and 96% specificity for FPLD in retrospective studies [21]. However, this method is not yet routinely implemented in clinical practice and is currently used in specialized or research settings. Computed tomography (CT) and MRI can also assess fat distribution, with the “Dunnigan sign” (hypertrophy of mons pubis fat with surrounding subcutaneous lipoatrophy) helping identify FPLD2. Additionally, a combination of gluteal fat thickness and the pubic/gluteal fat ratio from pelvic MRI has proven useful for diagnosing FPLD in women, as demonstrated in a Turkish cohort with predominantly European ancestry [22]. It is important to note, however, that this type of quantitative fat analysis is not routinely available in standard radiologic reports and generally requires evaluation by radiologists familiar with lipodystrophy or referral to specialized centers with expertise in body composition imaging. Lower-limb fat below the 1st percentile on DXA may suggest Dunnigan disease in women, particularly when accompanied by metabolic complications [23]. Unfortunately, the diagnostic value of anthropometric parameters for boys and adult males with FPLD2 has not been well-established due to the small sample size [24]. As mentioned earlier, race and ethnicity should be considered when evaluating normative versus disease cut-offs. The main advantages and disadvantages of the primary techniques used for quantifying body adipose tissue are summarized in Table 1.

**Table 1:** The main advantages and disadvantages of the primary techniques used for quantifying body adipose tissue

	Advantages	Disadvantages
Skinfolds	Simplicity, availability, low cost.	Lack of standardization. No quantification of intraabdominal (visceral) and ectopic fat.
DXA (dual energy X-ray absorptiometry)	Speed, non-invasive, easy-to-use method and lower costs compared to MRI.	Exposure to radiation (although low doses). Sensitivity to biological factors such as body fluid levels.
MRI (magnetic resonance imaging)	Ability to determine extra- and intramyocellular lipid content and evaluate solid organ characteristics (such as the liver) and bone marrow using spectroscopy.	More sophisticated and expensive compared to the other methods. Long time to determine fat distribution.

### Analytical Parameters

Fasting leptin and adiponectin levels can be informative, but various factors, such as breastfeeding, lactation, puberty, iron status, ketone bodies, specific fatty acids, and hormones, can influence adipokine levels [25–32]. Therefore, there are no standardized cut-offs for leptin and adiponectin levels to confirm or exclude the diagnosis of lipodystrophy, and they are not currently recommended as diagnostic tools [20, 33].

GL typically presents with very low or undetectable leptin levels, while patients with PL generally have low to normal leptin levels [33], except for those with MFN2-related FPLD, who exhibit very low leptin [34]. In addition to other clinical characteristics, measurement of high-molecular-weight (HMW) adiponectin may assist in differentiating GL from hypercatabolic or starvation states [35]. HMW adiponectin levels are extremely low in patients with GL, whereas in individuals with anorexia nervosa they are comparable to, or even higher than, those observed in normal-weight controls. This also applies to forms of GL associated with progeria [35]. Despite the generalized loss of adipose tissue, CGL2 is distinguished from other GL subtypes by higher adiponectin levels alongside suppressed leptin levels [36]. While these findings underscore the potential diagnostic value of adiponectin, particularly the HMW fraction, it is important to emphasize that this test currently plays a supportive, not primary role in clinical decision-making. Several limitations prevent its widespread adoption in routine practice, including its limited availability in standard clinical laboratories, the need for referral to specialized diagnostic or research facilities, and the absence of standardized reference thresholds.

In acquired partial lipodystrophy (APL), low serum complement C3, along with the presence of C3 nephritic factor and proteinuria [37], supports the diagnosis, while AGL may show low C4 levels [38, 39]. In patients with acquired lipodystrophy, the presence of autoantibodies can further support the diagnosis. The first fat-specific autoantibody targeting perilipin-1 has recently been discovered, present in 37–50% of patients with AGL [40–42]. Elevated creatine kinase levels are commonly observed in individuals with FPLD6 [43], as well as in those with laminopathies combined with muscular dystrophy or in individuals with concurrent juvenile dermatomyositis (JDM) and lipodystrophy.

### Genetic Testing

Confirmatory genetic testing is recommended in patients with suspected inherited lipodystrophy and should be considered for both the proband and at-risk family members. Although primarily indicated for congenital or familial forms, genetic testing may also be appropriate in selected cases of acquired lipodystrophy, particularly when the clinical presentation is atypical or shows features overlapping with known monogenic syndromes. In such scenarios, genetic testing can help rule out an underlying hereditary cause and guide diagnostic or therapeutic decision-making. However, it is important to note that new loci associated with genetic lipodystrophies are still being identified, so a negative result does not definitively exclude a genetic condition. Traditionally, genetic testing in lipodystrophy has been guided by phenotype, with molecular etiology-specific factors aiding in test selection and diagnosis [44]. However, this approach has several limitations. First, there is substantial clinical overlap among different lipodystrophy subtypes, which can obscure genotype-phenotype correlations and make it difficult to accurately prioritize candidate genes. Second, significant variability in clinical expression and incomplete penetrance within the same genetic subtype may further complicate phenotypic interpretation. Third, newly discovered genes and atypical mutations may not conform to classic diagnostic patterns, leading to missed or delayed diagnoses if testing is restricted to known phenotype-associated genes. For these reasons, broader sequencing strategies, such as multi-gene panels or chips, may prove to be more cost-effective and diagnostically efficient. Commercial panels, such as those offered by Blueprint Genetics, Invitae, and GeneDx, typically include the genes most commonly implicated in inherited forms of lipodystrophy (e.g., *LMNA*, *PPARG*, *AGPAT2*, *BSCL2*, *AKT2*, *PLIN1*, *CIDEC*, *LMNB2*, *MFN2*), and are regularly updated as new disease genes are discovered. Additionally, whole-exome and whole-genome sequencing are increasingly used in the diagnostic evaluation of lipodystrophy, with the potential to uncover novel disease mechanisms or clarify complex phenotypes. Although not yet routinely available in all clinical settings, these techniques can be accessed through specialized clinical genetics services, academic institutions, or certified commercial laboratories. Referral to such centers may be considered in complex or undiagnosed cases where broader genetic investigation is warranted. Genetic counseling is essential to determine the most appropriate test, navigate insurance coverage, and ensure proper communication of results.

### Screening for Comorbidities

All patients with generalized and partial lipodystrophy syndromes should be screened for comorbidities. In GL, DM often develops early and is resistant to standard treatments. Severe hypertriglyceridemia can lead to acute pancreatitis, occurring in 15–20% of GL cases [45, 46]. Liver disease may progress to steatohepatitis and cirrhosis [44], while macroalbuminuria (≥ 300 mg albumin/day) affects approximately 35–60% of GL patients. Other severe comorbidities include proteinuria, which can lead to renal failure, nephropathy and cardiovascular issues [47, 48].

PL is strongly associated with insulin resistance, hypertriglyceridemia, DM and low HDL levels [1], leading to an increased prevalence of metabolic syndrome in this population [3, 49]. PL is also associated with cardiovascular complications; notably, the prevalence of atherosclerotic vascular issues, such as coronary heart disease, is high among females with FPDL [50, 51]. High blood pressure is common and can be very severe in PL associated with pathogenic variants of *PPARG* [52–54]. Patients with APL may develop membranoproliferative glomerulonephritis and end-stage renal disease [55], while FPLD increases the risk of PCOS [56].

Based on these data, all patients with lipodystrophy should undergo comprehensive screening for DM, dyslipidemia, non-alcoholic fatty liver disease (NAFLD), and cardiovascular, renal, and reproductive dysfunction. The 2016 Multi-Society Practice Guidelines for the diagnosis and management of lipodystrophy syndromes outline the criteria and recommended frequency for screening associated comorbidities [1].

### Newer Diagnostic Tools

Because of the diagnostic complexity and phenotypic variability of lipodystrophy syndromes, innovative tools have been developed to support clinical decision-making. One such tool is LipoDDx^®^, a mobile application designed to assist physicians in identifying different subtypes of lipodystrophy, with an estimated diagnostic accuracy of approximately 80% [57]. The app also facilitates access to expert consultation and provides links to patient advocacy organizations. LipoDDx^®^ is available free of charge on both iOS (from the Apple Store, https://apps.apple.com/es/app/lipoddx/id1474797838) and Android (Google Play, https://play.google.com/store/apps/details?id=araujo.lipoddx) platforms, and it supports English, Spanish, and Galician languages.

### Treatment

While there is currently no cure for lipodystrophy, early intervention can significantly improve the morbidity and mortality associated with these conditions. Therefore, treatment should primarily focus on managing metabolic comorbidities.

### Lifestyle Intervention

Lifestyle intervention represents the cornerstone of lipodystrophy treatment. Although evidence supporting the benefits of specific diet patterns is limited, most patients are advised to follow a balanced diet consisting of 50–60% carbohydrates, 20–30% fats, and approximately 20% proteins. However, for those suffering from severe hyperglycemia and chylomicronemia-induced pancreatitis, a very low-fat diet with < 15% of energy from dietary fat may be beneficial. A recent case report from our group highlights the benefits of a very low-calorie diet (VLCD) in a patient with FPLD2 for improving metabolic and reproductive abnormalities [58]. All patients should avoid alcohol, as it can exacerbate hypertriglyceridemia and liver damage, especially since they often have a higher prevalence of non-alcoholic steatohepatitis [1]. In women, any estrogen therapy should be used with caution, since it may increase serum triglyceride levels. Managing carbohydrate intake is also crucial for patients with diabetes.

Consulting with dietitians familiar with these conditions can greatly aid in the management of these diseases [1, 26, 59]. When prescribing dietary treatments, setting a target weight is essential, with lower ideal weight targets recommended for all lipodystrophy forms. Dietary modifications can be particularly challenging in GL due to hyperphagia caused by leptin deficiency. Additionally, patients with GL may be misidentified as malnourished due to their extremely lean appearance and lack of subcutaneous fat. This may result in inappropriate nutritional interventions, such as force-feeding or high-calorie supplementation, which can further exacerbate metabolic complications including insulin resistance and hypertriglyceridemia. For patients with PL, maintaining an ideal weight with minimal ectopic fat accumulation is vital, potentially resulting in a BMI below 20 kg/m², as patients with FPLD often have a metabolic disease burden equivalent to a BMI 10 to 12 points higher than their measured value [60]. Given the high risk of atherosclerotic vascular disease, patients should avoid smoking and effectively manage hypertension. Regular physical exercise is encouraged to improve metabolic control, but physicians should assess for any heart or muscle abnormalities specific to the lipodystrophy subtype before initiating an exercise program.

### Treatments for Specific Comorbidities

Diabetes is common among patients with lipodystrophy, and management generally follows established national or international guidelines [61, 62]. Metformin is frequently used as a first-line pharmacological agent to decrease insulin resistance and improve glycemic control. Thiazolidinediones (TZDs) can enhance glycated hemoglobin (HbA1c), dyslipidemia, transaminases and hyperandrogenism in patients with PL [63, 64]; however they may increase regional fat deposits and lipomas [1, 65]. Furthermore, pioglitazone, a TZD, has been associated with an increased incidence of heart failure and fluid retention in patients with Type 2 diabetes (T2DM), and should be used cautiously [66]. The safety and efficacy of currently available TZDs in GL are not well established [1, 64]. Most TZD studies showed positive effects on liver function and steatosis, although a few have reported no effect [67].

Glucagon-like peptide-1 receptor agonists (GLP-1RAs) have shown promise in glucose-lowering therapy for FPLD, with reports of significant reductions in glucose levels [68]. In two case reports, HbA1c decreased by 2.7% and 4%, accompanied by weight loss of up to 30 kg observed [69]. Our recent retrospective study involving 14 patients with FPLD, compared to age- and sex-matched individuals with common T2DM, indicated that GLP-1RAs are effective and safe, leading to reductions in body weight, glucose, and triglyceride levels [70]. Nevertheless, their use in patients with a known history of pancreatitis or those at increased risk of pancreatitis should be approached with extreme caution. Additionally, although gradual tapering is not necessary, abrupt discontinuation of GLP-1 receptor agonists should be avoided, as it may result in a rapid rebound of hypertriglyceridemia and subsequent loss of metabolic control. This rebound can precipitate acute pancreatitis, particularly in individuals with a history of severe metabolic complications or prior episodes of pancreatitis.

Sodium-glucose cotransporter 2 inhibitors (SGLT2 inhibitors) may offer an additional therapeutic option, as they have shown effectiveness in reducing HbA1c levels in patients with PL, with a safety profile similar to that of patients with common T2DM [71]. However, individualized decision-making should consider the risks of peripheral vascular disease and ketoacidosis.

Due to severe insulin resistance, many patients with lipodystrophy require large doses of insulin to control their blood glucose levels. High daily insulin requirements may exceed the volume tolerated with standard U-100 insulin formulations, leading to injection site discomfort, delayed absorption, or poor adherence. In such cases, the use of concentrated insulin preparations, such as U-200 lispro, U-300 glargine, or U-500 regular insulin, can help minimize injection volume and improve both pharmacokinetic profile and patient comfort. U-500 is five times more concentrated than standard U-100 regular insulin and, although pharmacologically classified as regular insulin, its pharmacokinetic behavior is more similar to NPH insulin [72], allowing for twice- or three-times-daily administration. While randomized trials in lipodystrophy population are lacking, clinical experience and observational data suggest that concentrated insulin formulations may be particularly useful for delivering the required insulin amounts in patients with very high insulin requirements.

Only a few patients with lipodystrophy have been reported to have undergone bariatric surgery. Two patients had a pathogenic *LMNA* variant, two had a pathogenic *PLIN1* variant, while four patients were likely FPLD1 [73–77]. Most of these patients experienced improvements in metabolic parameters and weight loss following Roux-en-Y gastric bypass. However, further studies are needed to assess the long-term benefits of metabolic surgery in lipodystrophy patients.

Dyslipidemia management should align with general population guidelines [1]. Statins are commonly prescribed to reduce cardiovascular risk in conjunction with lifestyle modifications; however, these drugs must be used cautiously in patients with myopathy or muscular dystrophy [78]. Statin tolerance may be reduced in some individuals with FPLD, often due to muscle-related symptoms; however, robust evidence for increased susceptibility to statin-induced myopathy in this population is lacking, and this observation is primarily based on clinical experience. Fenofibrate may be added for insufficient triglyceride response, and while omega-3 fatty acids are widely used, their efficacy in this population remains unproven. New dyslipidemia medications, such as PCSK9 inhibitors, have not been systematically studied in lipodystrophy patients.

Many patients with lipodystrophy report experiencing anxiety and depression, along with chronic pain requiring multiple medications [4, 79]. Although the literature supporting these observations is limited, emerging data from international registries, such as the LD-Lync study (NCT03087253), provide valuable insights. A dedicated cohort study on partial lipodystrophy reported that 78.3% of patients experienced chronic pain, commonly attributed to conditions such as arthritis, lower back pain, fibromyalgia, or myopathy [4]. The mechanisms underlying this increased physical pain are likely multifactorial. The abnormal distribution or near absence of subcutaneous adipose tissue can result in reduced mechanical cushioning, leading to increased strain on muscles and joints. Furthermore, chronic systemic inflammation associated with severe insulin resistance, a hallmark of many lipodystrophic syndromes, may contribute to nociceptive and inflammatory pain. In some cases, central sensitization processes, such as those involved in fibromyalgia, may amplify pain perception. Psychological comorbidities, including depression and anxiety can further exacerbate the subjective experience of pain. Given these observations, patients should have access to psychological and mental health support. It is also crucial for them to receive appropriate care from a multidisciplinary team experienced in pain management.

Since physical discomfort or psychological distress in patients with lipodystrophy may stem from their appearance, they should also be given the option of undergoing cosmetic surgery [26]. Facial lipoatrophy can be improved with autologous fat transfer or dermal fillers [2, 80, 81] and excess adipose tissue from the face, neck, and vulvar region can be removed via lipectomy or liposuction [2]. Local injection of deoxycholic acid may also be considered for treating submental fat in certain FPLD subtypes [82].

### Leptin Treatment

Metreleptin, a human leptin analog, is the only approved treatment for lipodystrophy in the United States. The Food and Drug Administration (FDA) approved it for GL in 2014, with no lower age limit or metabolic disease threshold [83]. The European Medicines Agency (EMA) has approved it for patients with GL over 2 years of age and for those with PL over 12 years old who do not respond to conventional therapies [84]. Other countries including Japan, Canada, and Brazil, also approved leptin replacement therapy specifically for diabetes and/or hypertriglyceridemia in congenital or acquired lipodystrophy, both generalized and partial. In certain countries, patients may access metreleptin through compassionate use programs and other regulatory mechanisms.

#### Early Leptin Therapy Development

Our experience with metreleptin dates to 2000, following our attempts to obtain this therapy for patients in dire need, starting in 1997. By 1998, phase 2 trials demonstrated less than desired efficacy [85] and, in 1999, the Cambridge group reported the first report of leptin replacement in a child with congenital leptin deficiency [86]. Around the same time, the Brown and Goldstein laboratory reported encouraging results of leptin therapy in murine models of GL [87]. These developments collectively provided the rationale to initiate a clinical trial testing the efficacy of leptin replacement strategy in severe lipodystrophy. In the absence of any precedent for dosing in lipodystrophy, initial regimens were adapted from studies in congenital leptin deficiency, choosing to enroll patients with hypoleptinemia (females < 4 ng/dL and males < 3 ng/ml). In 2002, we reported that leptin replacement strategy resulted in 1.4% reduction in HbA1c, along with substantial decreases in background therapy, including the discontinuation of high doses of insulin. Additionally, there was more than a 50% reduction in circulating triglycerides, with some patients even stopping extensive treatments such as weekly plasma exchange [88]. These findings were subsequently confirmed in larger GL cohorts [89].

#### Leptin Therapy in GL

Metreleptin is particularly effective in GL, where endogenous leptin levels are extremely low. Since leptin regulates appetite through its effects on leptin receptors located in the hypothalamus [90], it has been reported that metreleptin therapy improves hyperphagia. This can lead to weight loss, although the weight typically stabilizes after several months [91–93]. In GL, metreleptin has also been shown to improve fasting glucose, lower HbA1c by 2% [93, 94] and reduce triglycerides by 60% [94] over a year. Additional benefits include decreased low-density lipoprotein cholesterol (LDL-C), total cholesterol [95, 96] and hepatic steatosis within 6–12 months [51, 93, 97–99]. Post-transplant use has also been reported to normalize hepatic histology [100].

Brown et al. demonstrated that metreleptin enhances peripheral and hepatic insulin sensitivity, reduces fasting glucose and triglycerides, and decreases liver fat content at least partly independent of its central effect on food intake [101]. Additional benefits of metreleptin in GL include attenuation of cardiac hypertrophy and improvement in septal e′ velocity [102], reduction in proteinuria and hyperfiltration [48], improvement in immunoregulatory function [103], normalization of gonadotropin secretion [104] and decrease in androgen levels in women with lipodystrophy [105].

#### Leptin Therapy in PL

Metreleptin therapy appears to be more effective in generalized than in partial forms of lipodystrophy and to induce more pronounced changes from baseline in patients with a more severe metabolic phenotype [94]. However, select patients with PL may also benefit from metreleptin, although the treatment effects are less pronounced and quite heterogeneous [106]. Early reports suggested that a lower baseline leptinemia may be associated with a better response [94]. However, an analysis of data across a wide range of leptinemia and fat loss demonstrated that a specific threshold of endogenous leptin concentrations in patients with PL is not a reliable predictor of clinical response to metreleptin therapy [107]. A recent study indicated that metreleptin treatment leads to similar metabolic improvements in patients with FPLD caused by *LMNA* and *PPARG* pathogenic variants. Baseline metabolic parameters, including HbA1c and triglyceride levels, appear to predict responsiveness to the therapy [108]. However, these findings are based on an open-label study and should be interpreted with caution due to the potential biases such as the Hawthorne effect or regression to the mean. To address these uncertainties, the FDA has mandated a global, placebo-controlled trial in PL, which is currently ongoing (NCT05164341).

#### Other Considerations for Leptin Therapy

Comparative evidence suggests that patients with GL and PL treated with metreleptin may experience up to a 65% reduction in mortality compared to metreleptin-naïve patients, despite having more severe disease [68]. However, confirmatory studies utilizing larger real-world and clinical trial datasets are needed. Additionally, metreleptin treatment has been reported to reduce the quality-of-life gap between untreated lipodystrophy and perfect health by 59% in GL and 31% in PL [68].

The effects of metreleptin on pregnancy, as well as its impact on labor and delivery, remain unknown in humans. While there are no systematic studies and metreleptin is not approved for use during pregnancy, there have been reported cases of pregnant individuals with lipodystrophy who were receiving metreleptin therapy, with no apparent signs of teratogenic effects [109]. However, it should be noted that most patients require additional insulin therapy especially in the third trimester of pregnancy despite metreleptin therapy.

Metreleptin therapy is generally well tolerated. The initial dose is 5 mg for women and 2.5 mg for men, with pediatric dosing adjusted according to weight. The dosage is adjusted based on metabolic responses and tolerance. Common adverse effects include injection site reactions and hypoglycemia, particularly if other antidiabetic treatments are not properly managed. Two significant adverse events associated with metreleptin are the development of neutralizing antibodies and T-cell lymphoma. The neutralizing antibodies may impair the biological activity of both exogenous and endogenous leptin, potentially worsening metabolic control [110–112]. T-cell lymphoma has been reported in patients with AGL treated with metreleptin [113]. However, the development of lymphoma has also been described in untreated patients with acquired lipodystrophy [113, 114], suggesting that the underlying autoimmune condition and immunodeficiency, rather than the therapy itself, may be the primary risk factor.

### Newer and Investigational Treatments

While current lipodystrophy treatments primarily focus on enhancing leptin signaling, other investigational approaches are being explored. A summary of new and investigational treatments, including registered studies from ClinicalTrials.gov, is provided in Table 2. While not included in the Table as a novel agent, we recently began planning prospective evaluation of dual incretin tirzepatide in patients with FPLD. The efficacy and safety of volanesorsen, a second-generation antisense inhibitor of human apolipoprotein C-III (apoC-III), has been evaluated in the BROADEN study (NCT02527343). This phase 2/3, 52-week, placebo-controlled trial involved patients with FPLD and concomitant hypertriglyceridemia and diabetes. Weekly subcutaneous volanesorsen (300 mg) reduced serum triglycerides by 88% and significantly decreased hepatic fat in 40 FPLD patients [115]. These results led to its approval in Brazil for hypertriglyceridemia in FPLD. A smaller NIDDK study (NCT02639286) showed improved lipoprotein lipase activity and enhanced peripheral insulin sensitivity [116]. While volanesorsen did not receive FDA approval for any indication in the U.S., a second-generation antisense inhibitor of ApoC-III, olezarsen (marketed as Tryngolza by Ionis Pharmaceuticals), has been developed. Olezarsen has demonstrated efficacy in familial chylomicronemia syndrome and is currently being investigated for severe hypertriglyceridemia. Although ongoing studies do not specifically target patients with lipodystrophy, they allow for their inclusion. Results from these studies are expected in the second half of 2025.

**Table 2:** Newer and investigational treatments for non-HIV related lipodystrophy syndromes

Drug	Mechanism of Action	Indication	Results/Notes	Sources
Volanesorsen	Antisense oligonucleotide targeting apolipoprotein C-III	FPLD	88% reduction in TG; approved in Brazil	[115, 116] ClinicalTrials.gov Identifier: NCT02527343 (Terminated) NCT02639286 (Completed)
Cholic acid	Endogenous ligand for FXR	LD	No reduction in hepatic TG content	[118] ClinicalTrials.gov Identifier: NCT00457639 (Completed)
Obeticholic acid	Selective FXR agonist	FPLD2	Data available on ClinicalTrials.gov	ClinicalTrials.gov Identifier: NCT02430077 (Completed)
Vupanorsen (AKCEA-ANGPTL3-LRx)	Antisense angiopoietin-like 3 protein (ANGPTL3) inhibitor	FPLD	Positive effects on lipid profiles reported	[119] ClinicalTrials.gov Identifier: NCT03514420 (Completed)
Gemcabene	Lipid metabolism modifier	FPLD	Data available on ClinicalTrials.gov	ClinicalTrials.gov Identifier: NCT03508687 (Completed)
Baricitinib	JAK1/JAK2 inhibitor	Autoinflammatory syndromes	Improvement in clinical manifestations	[120] ClinicalTrials.gov Identifier: NCT01724580
REGN4461 (Miba)	Monoclonal antibody activating LEPR	GL and PL	Good tolerability; good results in patients with undetectable leptin	[121] ClinicalTrials.gov Identifier: NCT04159415 (Completed) NCT05088460 (Terminated) NCT04710056 (Ongoing)
Setmelanotide	Melanocortin 4 receptor agonist	Single patient with partial LD associated with leptin deficiency and multiple autoimmune diseases	Not significant metabolic benefit	[122] ClinicalTrials.gov Identifier: NCT03262610 (Completed)
Pegvisomant	Recombinant growth hormone receptor antagonist	PL	Not yet available; trial ongoing	ClinicalTrials.gov Identifier: NCT05470504
Cyclophosphamide	Antineoplastic alkylating agent	Associated acquired LD with T1DM	Not yet available; trial ongoing	ClinicalTrials.gov Identifier: NCT03936829

^a^FPLD=familial partial lipodystrophy; TG = triglycerides; FXR = farnesoid X receptor; LD = lipodystrophy; GL = generalized lipodystrophy; PL = partial lipodystrophy: T1DM = Type 1 Diabetes Mellitus

In addition to the listed approaches in Table 2, one promising avenue is gene therapy. A recent study utilizing adeno-associated virus (AAV) gene therapy in adult seipin knockout mice demonstrated significant restoration of white adipose tissue development and improvement in metabolic health in this model of CGL [117]. This suggests that gene therapy may address various metabolic complications in patient with congenital lipodystrophy. Furthermore, advancements in genetic editing strategies offer hope for potentially curing many genetic diseases.

## Conclusions

Lipodystrophy syndromes encompass a range of diseases characterized by inadequate fat storage and function, leading to various metabolic disorders. Clinical acumen and awareness are crucial for accurate diagnosis, although more objective criteria are being developed by research groups in this field. Leptin therapy has emerged as a breakthrough, particularly in GL, where it has shown the greatest impact. In PL, strategies designed to align excess fat storage demands with adipose tissue size, by addressing low BMIs and promoting weight loss through novel agents like incretin therapy, show promise until curative approaches targeting the underlying specific defects can be developed. As we approach individual patients, it is essential to adopt a compassionate, multidisciplinary approach that addresses the multisystemic nature of the disease, with particular attention to challenges such as hyperphagia, pain, and fatigue.

## Key References

- Adiyaman SC, Altay C, Kamisli BY, Avci ER, Basara I, Simsir IY, et al. Pelvis Magnetic Resonance Imaging to Diagnose Familial Partial Lipodystrophy. J Clin Endocrinol Metab. 2023;108(8):e512-e20. 10.1210/clinem/dgad063
-
  - The combined use of gluteal fat thickness and pubic/gluteal fat ratio from pelvic MRI is a promising method to diagnose Familial Partial Lipodystrophy (FPLD) in women.
- Corvillo F, Abel BS, López-Lera A, Ceccarini G, Magno S, Santini F, et al. Characterization and Clinical Association of Autoantibodies Against Perilipin 1 in Patients With Acquired Generalized Lipodystrophy. Diabetes. 2023;72(1):71-84. 10.2337/db21-1086.
-
  - Anti-Perilipin 1 autoantibodies can be a useful biomarker for the diagnosis of certain forms of Acquired Generalized Lipodystrophy.
- Foss-Freitas MC, Besci Ö, Meral R, Neidert A, Chenevert TL, Oral EA, et al. A Very-Low-Calorie Diet Can Cause Remission of Diabetes Mellitus and Hypertriglyceridemia in Familial Partial Lipodystrophy. Obes Facts. 2024;17(1):103-8. doi: 10.1159/000533992.
-
  - The use of a monitored Very-Low-Calorie Diet can be useful for patients with metabolic and reproductive abnormalities due to loss of adipose tissue and abnormal distribution.
- Foss-Freitas MC, Imam S, Neidert A, Gomes AD, Broome DT, Oral EA. Efficacy and Safety of Glucagon-Like Peptide 1 Agonists in a Retrospective Study of Patients With Familial Partial Lipodystrophy. Diabetes Care. 2024;47(4):653-9. 10.2337/dc23-1614.
-
  - This is the first and largest data set that demonstrates GLP-1RA is a safe and effective treatment for patients with Familial Partial Lipodystrophy, offering benefits in lowering HbA1c, weight, and BMI.
- Bansal R, Cochran E, Startzell M, Brown RJ. Clinical Effects of Sodium-Glucose Transporter Type 2 Inhibitors in Patients With Partial Lipodystrophy. Endocr Pract. 2022;28(6):610-4. 10.1016/j.eprac.2022.03.006.
-
  - SGLT2i use may lead to better glycemic control in a subset of patients with partial lipodystrophy and severe insulin resistance. SGLT2i can be considered as part of the armamentarium for management of these patients after careful consideration of risks and benefits.
- Oral EA, Garg A, Tami J, Huang EA, O'Dea LSL, Schmidt H, et al. Assessment of efficacy and safety of volanesorsen for treatment of metabolic complications in patients with familial partial lipodystrophy: Results of the BROADEN study: Volanesorsen in FPLD; The BROADEN Study. J Clin Lipidol. 2022;16(6):833-49. 10.1016/j.jacl.2022.08.008.
-
  - The BROADEN study demonstrated that volanesorsen significantly reduced serum triglyceride levels and hepatic steatosis in patients with Familial Partial Lipodystrophy.
- Altarejos JY, Pangilinan J, Podgrabinska S, Akinci B, Foss-Freitas M, Neidert AH, et al. Preclinical, randomized phase 1, and compassionate use evaluation of REGN4461, a leptin receptor agonist antibody for leptin deficiency. Sci Transl Med. 2023;15(723):eadd4897. 10.1126/scitranslmed.add4897.
-
  - This translational work indicates that the LEPR agonist mAb REGN4461, also known as Mibavademab, could provide therapeutic benefits for conditions associated with leptin deficiency or hypoleptinemia. The paper shows data from the first in-human obesity study as well as a compassionate use experience in a patent with atypical partial lipodystrophy who developed neutralizing antibodies to metreleptin. Preclinical data presented include rodent models of leptin deficiency and generalized lipodystrophy.

## Data Availability

No datasets were generated or analysed during the current study.
